# Searching for answers in an uncertain world: Meaning threats lead to increased working memory capacity

**DOI:** 10.1371/journal.pone.0204640

**Published:** 2018-10-03

**Authors:** Daniel Randles, Rachele Benjamin, Jason P. Martens, Steven J. Heine

**Affiliations:** 1 Department of Psychology, University of Toronto, Toronto, Ontario, Canada; 2 Department of Psychology, University of British Columbia, Vancouver, British Columbia, Canada; 3 School of Social Sciences, Birmingham City University, Birmingham, England; Vrije Universiteit Brussel, BELGIUM

## Abstract

The Meaning Maintenance Model posits that individuals seek to resolve uncertainty by searching for patterns in the environment, yet little is known about how this is accomplished. Four studies investigated whether uncertainty has an effect on people’s cognitive functioning. In particular, we investigated whether meaning threats lead to increased working memory capacity. In each study, we exposed participants to either an uncertain stimulus used to threaten meaning in past studies, or a control stimulus. Participants then completed a working memory measure, where they either had to recall lists of words (Studies 1, 2), or strings of digits (Studies 3, 4). We used both a frequentist approach and Bayesian analysis to evaluate our findings. Across the four studies, we find a small but consistent effect, where participants in the meaning threat condition show improved performance on the working memory tasks. Overall, our findings were consistent with the hypothesis that working memory capacity increases when people experience a meaning threat, which may help to explain improved pattern recognition. Additionally, our results highlight the value of using a Bayesian analytic approach, particularly when studying phenomena with high variance.

## Introduction

For the most part, our worlds unfold as we expect. It rarely snows in the summer, fire tends to be hot, generally our friends don’t try to hurt us, and when we go to bed at night, we expect to wake up in the morning. But on occasion things may happen that don’t make so much sense. A variety of theoretical perspectives have emerged to account for how people react when these unexpected events occur (for reviews see [1–3]). In particular, the Meaning Maintenance Model (MMM; [1,4]) proposes that people have a need to maintain a sense of meaning. The “meaning” in this model refers to expected relations–that is, the ideas that we can connect to any cognition, emotion, or behaviour. So, for example, what one’s alma mater “means” to someone is all the ideas that they can relate to it–their memories of friends, classes, the school’s reputation, the opportunities that it afforded, parties, the food in the dining hall, and so on. If any of these relations changed, then so would one’s perceived meaning of their school. Moreover, if some dramatic unexpected event were ever to happen at one’s school, such as a school shooting, or an embarrassing scandal, then people might experience a “meaning threat,” as they would struggle to integrate this new piece of information that is at odds with their existing understanding of their school.

There are a variety of experiences that can constitute meaning threats. For example, the experience of interpersonal rejection entails the severing of relationships between people [5,6], encounters with perceptual anomalies suggest that the world is different than one understands [7,8], surrealist art juxtaposes contradictory elements together in unfamiliar ways [9,10], feelings of personal uncertainty or cognitive dissonance diminish one’s confidence in one’s meaning frameworks [11–14], an awareness of conflicting attitudes undermines a sense of order [15], feelings of a lack of control deprives one from the sense that one’s actions impact the world [16,17], and reminders that one will some day die makes one consider how all the relations that they have with the world and others will someday inevitably come to an end with their death [18–20]. Meaning threats can result from a vast variety of situations and experiences.

### Responses to meaning threats

The MMM maintains that people seek to remain in a state of homeostasis where the world appears to them in ways that are consistent with their expectations. When people encounter events that are unexpected or hard to process, they experience some unconsciously perceived aversive arousal that prompts them to restore a feeling that the world makes sense again [21]. A variety of different palliative responses to restore meaning have been identified. One response is to assimilate the anomaly such that it no longer seems anomalous [22–24]. People may preserve their existing meaning frameworks by assuming that the encountered anomaly is not anomalous at all, such as how a black queen of diamonds might appear to actually look red [25], or that an innocent person beset by a horrible tragedy may be seen as somehow deserving it, thereby preserving a belief in a just world [26]. A second commonly documented response to encounters with the unexpected is that people may accommodate their meaning frameworks, by modifying their understanding of the world to take into account the anomalous event [22,27]. For example, after agreeing to help an experimenter by telling the next participant that a really boring task was actually quite interesting, one might alter their meaning frameworks to convince themselves that they actually enjoy mindless, repetitive tasks [28], or upon learning that ingesting a bacterium causes an ulcer a doctor may revise her existing theory about the nature of ulcers (see [29]). Theories of assimilation and accommodation have been common in many different accounts of meaning (e.g., [22–24,27,30]); however, these responses to unexpected events each have their respective shortcomings. Assimilation is often not complete–for example, even though participants might not be able to consciously notice that a set of playing cards includes reverse-colored cards, they still show evidence that the anomalous cards are bothersome to them [8]. And accommodation can be cognitively demanding—when people are presented with evidence that challenges their understanding of the world, it is hard for them to rethink their entire worldview [31] but it is potentially easier for them to dismiss the evidence outright. Hence, in the immediate aftermath of an encounter with an anomaly, people may not have the ability to completely assimilate or accommodate the meaning threat.

Given the limits of assimilation and accommodation in resolving any discovered anomalies, the MMM has explored other psychological reactions to unexpected encounters that go under the broad rubric of fluid compensation [32,33]. When faced with an anomaly that can’t be fully assimilated or accommodated, people may instead compensate through an entirely separate palliative process that serves to dispel the unpleasant arousal caused by the perceived meaning threat. The most studied of these is affirmation. That is, when people have detected a shortcoming in a meaning framework they may increase their commitment to another, entirely separate, meaning framework [1]. Though this does nothing to resolve the original offending anomaly, it does allow the individual to regain a general sense of meaning. There are many examples of affirmation in the literature across a broad array of different theoretical paradigms. Dozens of studies from the terror management literature find that when people contemplate their own mortality they subsequently engage in cultural worldview defense, by which they increase their commitment to their beliefs about the world [34]. When people are made to feel uncertain, they subsequently engage in more intergroup discrimination (e.g., [35]). When people act in a manner dissonant with their attitudes, they will show enhanced polarization of unrelated attitudes towards affirmative action [13]. Or when people read a short story by Kafka that violates their expectations, they come to identify more with their culture [9]. All of these various findings cohere in revealing increased commitment to previously held beliefs following an encounter with a meaning threat.

Studies of affirmation share one feature in common: following a threat, participants are provided with an alternative meaning framework that they can affirm. However, what happens if participants are not provided with any such alternative framework? A number of studies find evidence that when people feel uncertain they exhibit heightened attentional vigilance for new information [17,36–38]. Moreover, some studies have found that people show a heightened ability and/or motivation to search for patterns in the environment, in an effort to discover new meaningful relationships (e.g., [15,17]). This form of threat compensation has been termed abstraction [4,38].

Some evidence for abstraction comes from Proulx and Heine [38] who observed that after reading a surreal short story by Franz Kafka, participants performed better on an implicit grammar learning task compared with those who read a control story. Without knowing that they were doing so, people attended more to the rules of the artificial grammar following the surreal story, enabling them to later identify letter strings that conformed to the grammar. In a follow-up study, Randles et al. [39] showed that even when a threat went undetected (in this case, participants were subliminally presented with incoherent word pairs), participants were still better able to learn an artificial grammar than when presented with coherent word pairs. Although abstraction seems to fit within the MMM’s framework of ‘meaning-lost, meaning-restored’ [4], much of how it works remains poorly understood. One possibility is that when people are made to feel uncertain, they are more prepared to make sense of a changing environment. They should be in a heightened state of arousal as they try to make sense of what is happening around them. To the extent that this is the case, we would expect that uncertainty would prompt temporary increases to working memory capacity. This paper describes studies designed to test this hypothesis.

### Error evaluation, conflict detection, and meaning

One way to understand the mechanisms underlying abstraction is to consider what we know of the brain systems that handle cognitive conflict. Converging lines of neuroscience research reveal that the anterior cingulate cortex (ACC) responds to detected conflicts or errors in processing [40,41]. Though there is widespread disagreement about the specific role of the ACC, which may be implicated in a variety of other cognitive or affective processes that go beyond our current focus—for example, pain [42], social pain [43] distress more generally [42,44,43], and others (see, e.g., [45,46,47])—there is firm evidence that the ACC is activated by conflict monitoring [48,2]. Specifically, when people perform complex tasks, the ACC triggers a series of responses in the prefrontal cortex (PFC) that lead to greater executive functioning [49]. The two systems work in concert to help in the detection and correction of processing errors, with the ACC performing a conflict monitoring role and the PFC performing a cognitive control role [40]. This signal appears to enhance cognitive control, as the strength of ACC activation in a preceding trial predicts reduced reaction time and errors on a subsequent trial, as well as reduced ACC activation and increased activation of the prefrontal cortex (a region associated with cognitive control; [50]). In other words, detecting an anomaly that leads to error triggers greater control and greater expectation that anomalies will occur, which in turn reduces both ACC activation in response to anomalies and the likelihood of making an error. This is the process that we speculate is most at play during abstraction, though we acknowledge that meaning threats produce a variety of other responses (for example, affirmation) that may also result from activation of this neural region; indeed, much of the threat defense literature agrees that anomalies elicit anxiety, or other negatively-valenced experiences (see [2,12]) and often cite the ACC as the origin of this response (e.g., [51,12,2]).

Research from a variety of different paradigms reveals that encounters with meaning threats lead to greater activation in the ACC (for reviews, see [2,4]). For example, studies find increased activation in the ACC when people encounter inconsistencies that arise either through cognitive dissonance [52,53] or behaving at odds with one’s self-concept [54]. Likewise, when people are led to consider how they are going to die someday–perhaps, the ultimate meaning threat [20,55]–they similarly show enhanced ACC activation [56].

In addition, some converging evidence for the similarity in neural responses to various kinds of meaning threats comes from research where participants ingest either a painkiller, such as acetaminophen, or a placebo. After consuming a painkiller participants show less activation in the ACC following interpersonal rejection [57] or when making errors in an Error-related Negativity paradigm [58]. Likewise, consuming painkillers leads to weaker defensive reactions to mortality salience and uncertainty manipulations [10], as well as less dissonance reduction [59]. The latter effects are theorized to arise from the diminished ACC activation following the consumption of painkillers.

Taken together, these studies indicate that a variety of meaning threats lead to heightened ACC activation. We suggest that this activation increases people’s propensity to attend to events in their environment. Indeed, more general principles of threat defense also support our supposition that expectancy-violating events elicit attentional control. A long-standing concept in biopsychology is the behavioral inhibition system (BIS), which is theorized to manage the anxiety and avoidance that accompanies conflict detection [60,61]. The BIS is activated when there is a threat that causes people to move from a state of approach to anxiety and risk assessment [62,63]. It is believed to rely on activation in the ACC [64] as well as neural substrates associated with anxiety like the amygdala and septo-hippocampal system [60,65,66]. Activation of the BIS is associated with arousal in response to negative or potentially life-threatening events, which in turn leads people to pay more attention to their environment [60,67]. However, it has been proposed that the BIS is activated by surprising or uncertain stimuli, in addition to negative stimuli [60]. Therefore, we posit that meaning threats produce BIS activation, which in turn leads people to engage in greater attentional control.

Given that ACC activation has been found to predict executive functioning [50,68,69], and given that theories of the BIS suggest that conflict detection is associated with increased vigilance [60], it follows that meaning threats might lead people to engage in more careful processing of stimuli in their environment. We sought to test this hypothesis by measuring performance on tasks that measure executive functioning.

### Working memory capacity and cognitive control

One core executive function is working memory, the cognitive process associated with holding information in mind and manipulating it [70,71]. The prevailing view is that working memory includes both a storage component and an attentional control component [72–74]. It is this second component that leads us to believe that working memory may be one of the resources recruited when managing uncertainty.

The attentional control component of working memory, referred to as the central executive, is what allows individuals to stay focused on task-relevant information and selectively ignore task-irrelevant information [75]. Investigations of the constructs underlying working memory capacity (e.g., [74]) as well as neural imaging studies (reviewed in [75]) suggest that conflict detection and conflict resolution are critical features of working memory capacity. Indeed, the ability to suppress competing information is essential to performance on working memory tasks, which typically involve completing two activities simultaneously and switching attention between them (see [76]). Furthermore, there is general agreement that the ACC—the area of the brain most associated with meaning threats—is implicated in the aspect of working memory that involves suppressing competing information [77]. Therefore, stimuli that make people feel uncertain may activate the same conflict resolution process that is activated during working memory tasks.

The MMM is not the first model to forward a hypothesis about the effect of threat on attention. Among them is the Unconscious Vigilance Model (UVM; [37]) such that individuals experience heightened reactivity to affective targets after experiencing a discrepancy. This heightened vigilance is not theoretically related to motivations like relieving anxiety, but simply facilitates appropriate responding to potentially threatening events [37, 2]. Though it may follow from the UVM that working memory capacity increases after a discrepancy under some circumstances, this model has no explicit prediction about people’s responses to targets that are not affectively charged. Jonas et al. [2] proposed a more general model of threat defense, suggesting that the mechanism by which individuals respond to threat is through the behavioral inhibition system (BIS), which is activated during the initial discrepancy detection, and is followed by approach-oriented behavior mediated by the behavioral activation system (BAS). Like the MMM, this model predicts that threats can increase accuracy in information processing, and that this represents a general increase in vigilance rather than targeted efforts to resolve the threat.

There are also models that may lead to the opposite prediction: that uncertainty decreases working memory capacity. For example, stereotype threat, which according to some characterizations originates from a conflict between self-schemas, decreases working memory capacity when individuals are required to engage in task-relevant behaviour (see [78,79]). On the other hand, we are not predicting that uncertainty makes people more focused on task-relevant problems. The predictions that derive from the MMM are relevant to people’s global processing, rather than their capacity to remain focused on the task at hand. In fact, there is evidence suggesting that when the source of uncertainty does not resolve itself quickly, uncertainty can draw attention away from the present goal and towards more distal goals (e.g., [80]) which is theorized to explain people’s tendency to affirm unrelated schemas when more proximal strategies are unsuccessful (see [2]). For this reason, we cannot claim that uncertainty always enhances people’s ability to solve problems. Depending on the problem of interest, it may actually inhibit this ability. The current topic of interest is how working memory generally increases, rather than specific targeted efforts to resolve the source of uncertainty.

Based on current evidence from research in uncertainty and cognitive control, we hypothesize that threats to meaning result in greater executive functioning, and specifically, increased working memory capacity. This may lend some further context to the finding that pattern learning increases following a meaning threat. Furthermore, it would be consistent with the claim that the ACC and PFC are recruited to resolve uncertainty. We propose that uncertainty triggers a series of responses that lead to increased working memory capacity and more effortful thinking.

In the following sections, we outline our results using Bayesian statistics as well as a more traditional frequentist approach. One benefit of Bayesian analysis is that it allows us to test whether there is good evidence for the null hypothesis, in addition to the alternative hypothesis. A traditional frequentist approach does not allow researchers to determine whether their findings support a null hypothesis. This affects both the accuracy of the inferences people draw from their findings, and their likelihood of establishing a point estimate of the true effect size if one exists [81].

Bayesian statistics are especially useful for updating information with more data, producing cumulative evidence for a model [82]. For this reason, Bayesian statistics empower researchers to correctly interpret failures to replicate [83,84]. Not only are p-values more likely to produce significant findings when the null is true; they also are likely to produce nonsignificant results despite that there is a true effect [85]. Bayesian analysis is particularly well-suited to the present research because of the many conceptual and direct replications we conducted. This presents us with a unique opportunity to estimate the size of our effect using Bayesian statistics, evaluating support for our theoretical perspective as well as support for the null.

## Materials and methods

### Study 1

This research was granted approval by the University of British Columbia Office of Research Services Behavioural Research Ethics Board. The approval code for this research is H09-02437. Written consent was obtained for studies conducted in-lab, and for studies conducted online over Amazon's Mechanical Turk, consent was obtained in the form of a checked box.

Participants were undergraduate students who volunteered in exchange for course credit (*N* = 107). Mean age was 19.89 (*SD* = 4.03), sample was 80.4% female, 54.2% East Asian, 22.4% European ancestry, and 23.4% other cultural backgrounds. The study took place on a computer, where participants first completed a meaning threat as the manipulation, followed by the working memory measure.

#### Sensible-senseless word priming

This task was designed to subliminally present participants with word-pairs that they had never seen before, and that violated common rules of language, such as Magic-Softly. While this inconsistency should be perceived as a threat to meaning, it is also likely easily resolved, so word-pairs were presented at near subliminal exposures. This task has previously been shown to cause compensatory affirmation and improved ability on an implicit pattern-learning task [39].

#### Working memory measure

The working memory task was taken from Schmader et al. [78]. Participants were told they would be given single words, which they would need to remember and recall after a number of trials. They would also be shown sentences, where they would need to count and report the number of vowels. Participants completed these alternating trial cycles for 4 to 6 repetitions, after which point they would be asked to recall all the single words, and then forget them for the next round. There were 12 rounds with 60 trial-pairs in total. Participants were scored on the proportion of single words correctly remembered. Across all studies, participants were excluded from our analyses if they took 10 minutes or under to complete the working memory task, or if they took over 30 minutes. For online studies, we also included a quality check to ensure that participants were not writing down the number strings. This was a 12-digit number that participants would not be able to recall with memory alone. Participants who were able to correctly respond to this question were excluded from our analyses.

#### Procedure

Participants first provided written consent using either a physical consent form for studies conducted in-lab, or a digital consent form for studies conducted on Mturk. They were then told that they would see a number from 1–9 (excluding 5) and would then be asked whether the number was even/odd or high/low. For each trial, a fixation cross was presented for 1000ms, followed by the number for 356ms, a randomly jittered blank space for 400-700ms, the subliminal stimulus window of 30ms, a 200ms static block meant to serve as a backwards mask, and finally the participant’s question concerning the number. Participants in the control condition were presented with no subliminal stimulus for the first ten trials, followed by 20 trials of sensible word-pairs (e.g. Cheese-Cake), then a 2^nd^ set of 30 trials following the same order. The meaning threat group received the same stimuli, except that trials 21–30 and 51–60 contained senseless word-pairs (e.g. Bull-Left). Senseless word-pairs were created by recombining the sensible pairs presented in the control condition. Scripts to run the experiment in Inquisit are available in the SOM.

### Study 2

Study 2 is a conceptual replication of Study 1. We changed our participant pool to Amazon’s Mechanical Turk (Mturk) to gather a larger sample (*N* = 431). Mean age was 33.55 (*SD* = 11.91), sample was 64.4% female, 80.0% White, 5.3% Black or African American, and 13.6% other ethnicities.

We changed the meaning threats to include both a mortality salience condition, and a "reversed cards" condition. The former involved writing about death, while the latter involved playing blackjack online, where halfway through some of the suit colors on the cards are flipped (red to black or black to red). We also included a condition where participants experienced both meaning threats. Additionally, we increased the difficulty of the working memory task. This was done because exploratory analysis of the DV in Study 1 indicated most people answered the earliest and easiest questions perfectly, with very little variation between groups.

### Study 3

Participants were students who volunteered in exchange for course credit (*N* = 174). Mean age was *M* = 20.86 (*SD* = 3.91), sample was 83.9% female, 47.1% East Asian, 24.7% White, 12.6% South Asian, and 15.6% other cultural backgrounds.

Study 3 uses the same manipulations as Study 2, but we introduced a new DV. After the manipulation, participants are given strings of digits that they must remember and type back in backwards. For example, a participant might be presented with 4–6–3–5–6, and would need to type 6–5–3–6–4 [86]. There were 18 trials of this task, and responses were scored according to the proportion of correct answers participants provided. Digits are presented one at a time with accompanying audio. This study was run in-lab with undergraduate student participants.

### Study 4

Study 4 is a direct replication of Study 3 using an Mturk sample (*N* = 348). Mean age was *M* = 33.3 (*SD* = 11.3), sample was 62.2% female, 79.0% White, 7.2% Latin, and 13.8% other cultural backgrounds.

## Results

### Study 1

We analyzed the data across our studies using two distinct approaches. First we present the conventional approach, regressing score onto condition (analogous to a t-test). The second approach involves a Bayesian analysis, where we estimate the distribution of the posterior likelihood for the effect size, based on initially relatively flat priors but updating through the studies. The dependent variable is standardized for analysis, making it easier to compare models across studies and update the prior distributions for the Bayesian analysis moving forward.

**Fig 1:**
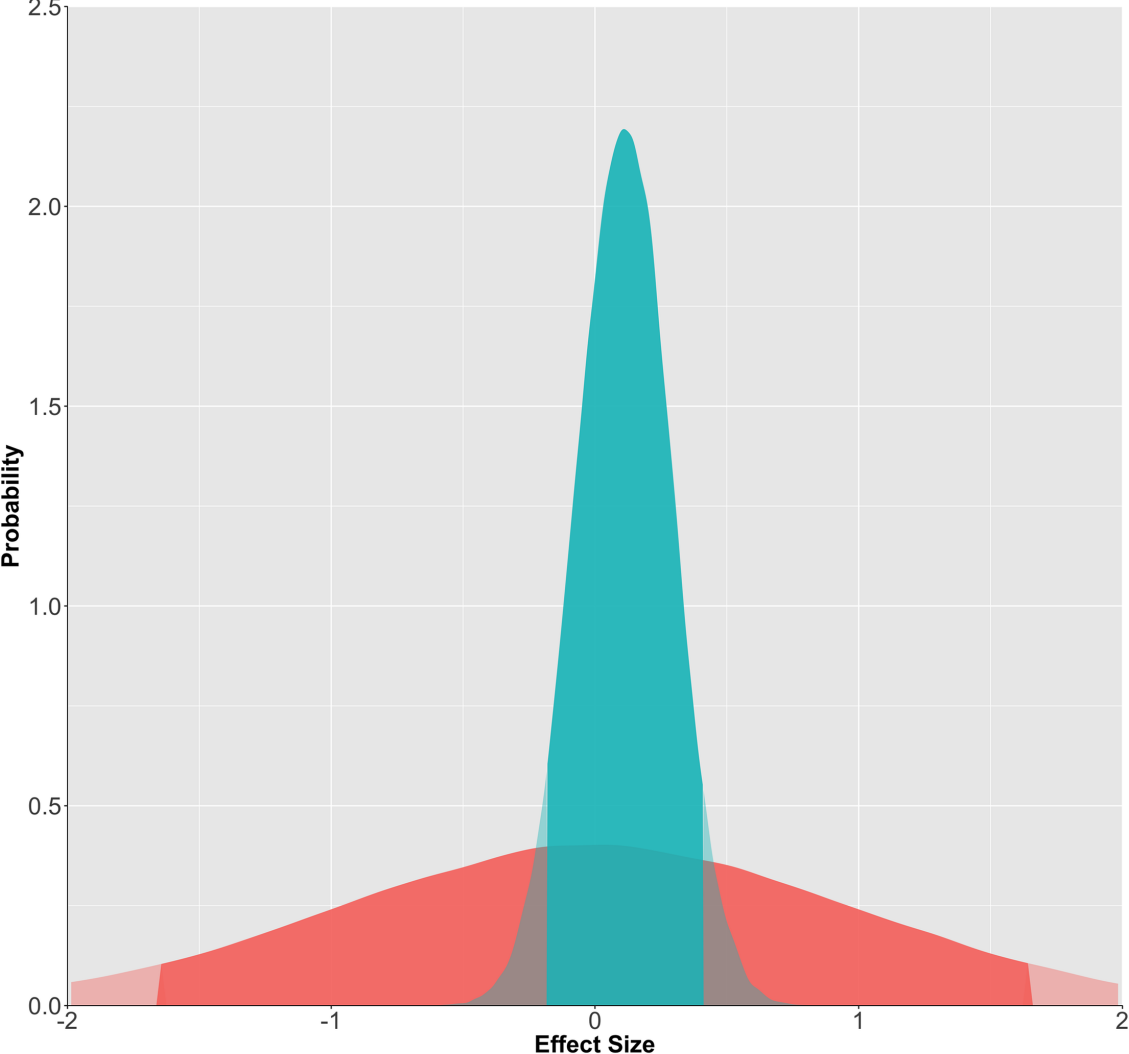
Prior and posterior distribution of the effect size. Red distribution is the prior probability of the effect, green is the posterior distribution which accounts for study data. Solid region represents the 95% probability window, shaded regions are outside this window. Results of study 1 indicate that effects larger than .6 are very unlikely. There is still high uncertainty regarding whether the true effect size is zero, or small but decidedly non-zero.

Total sample size was *N* = 107 (control = 51, threat = 56; no participants were removed). Control group mean score and *SD* are .68 (.17), meaning threat group values are .72 (.14). The conventional statistical test for condition, *B* = .25[-.13, .62], *p* = .21, indicates failure to reject the null. For the Bayesian analysis, we assigned priors as follows: the intercept was defined with a mean based on the normal distribution, and a standard deviation uniformly distributed from 0–2. These priors reflect our knowledge of the mean and standard deviation (since the data have been normalized). The prior estimate of the effect for condition was normally distributed around 0 with a *SD* of 1, implying that the effect lies somewhere within a *d* +- 2; a sensible opening assumption for behavioral experiments given that most effects would not lie outside of this range. The prior is slightly biased towards a *d* = 0, but is flexible enough that it is essentially flat for most reasonable values. Using the "rethinking" package in R [87], we ran a Bayesian regression model, and found a similar effect, *B* = .23[-.14, .60]. As a first study, these results are inconclusive, with both approaches yielding similar interpretations (see Fig 1). Moving to study 2, however, we have stronger expectations for the effect, namely that it is either zero, or that if it exists, it is likely small. We can update our priors for the next study by simulating a posterior distribution based on our expectations. This new prior is thus somewhat akin to a directional test, in that the model is biased against negative effects. However, it is also biased against effects larger than about .60, and in exchange is somewhat biased in favor of seeing a small but positive effect as more likely.

### Study 2

Sample size, mean, and standard deviation for each group on the working memory task were as follows: Control *M* = .72, *SD* = .19, *n* = 104; mortality salience *M* = .74, *SD* = .20, *n* = 112; cards *M* = .73, *SD* = .17, *n* = 90; both meaning threats *M* = .78, *SD* = .16, *n* = 125. Twenty-three participants were removed because of technical problems, because they failed one of our various quality checks, or because admitted cheating on the working memory task in the debriefing, or because they noticed the color-reversed playing cards in the blackjack game. Though 55 participants indicated that they noticed something unusual about the blackjack game, only 3 people pointed to the card color as the unusual event. Specifically, they responded "some symbols were not the usual color", "the suites", and "changed colors is all and I lost at lot". Most other comments were an attempt to explain the users’ particular results, identifying that they won or lost more than they should have, and suggesting either that the dealer cheated or their betting pattern affected the result (none of which was the case).

As with study 1, we present both the conventional frequentist and Bayesian analysis. For the frequentist approach, we ran a single regression model, with the intercept at the control condition and each experimental condition dummy coded separately. The effects for condition are small and mostly non-significant: mortality salience *B* = .10[-.17, .36], *p* = .48; cards *B* = .07[-.22, .35], *p* = .65; both threats *B* = .31[.05, .57], *p* = .02. From a frequentist perspective, these results are quite deflating, but they shouldn't be. All three effect-size point-estimates are within a sensible range, given our expectations for the true effect size (i.e. somewhere between -.20, and .60). A Bayesian analysis that estimates the effect in the context of our expectations will tell a slightly different story.

**Fig 2:**
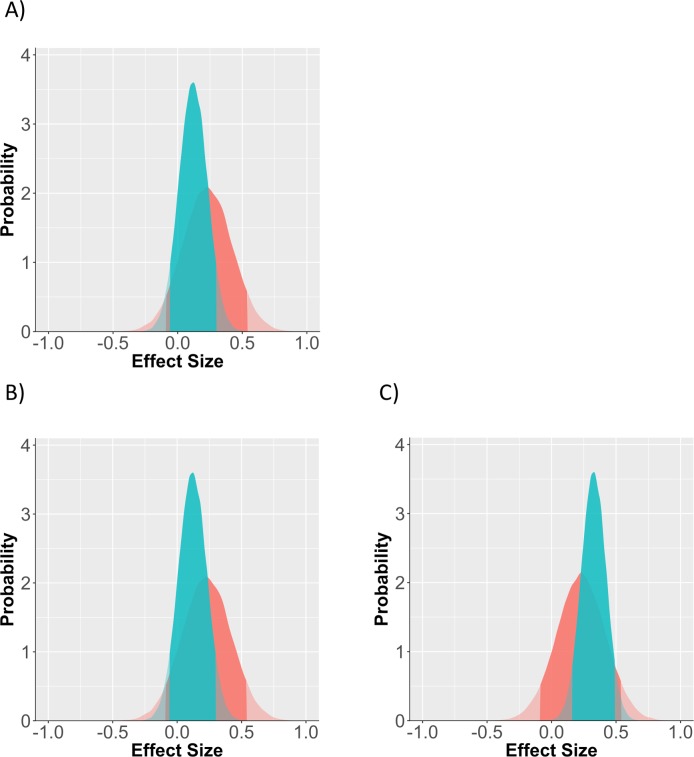
Prior vs posterior distribution for each of the 3 conditions. **(**A) mortality salience, (B) reverse cards, (C) both manipulations. Red distribution is the prior probability of the effect, green is the posterior distribution which accounts for study data. Solid region represents the 95% probability window, shaded regions are outside this window.

We used the same relatively flat priors for the mean and standard deviation of the sample, but updated our estimate of the effect to *M* = .23, *SD* = .19. Results offer a similar interpretation, in that we are only confident the double meaning threat condition produced a non-zero effect (See Fig 2 for prior and posterior distributions and, see Table 1 for parameter estimates; the interpretation is similar to the conventional analysis). However, because we were willing to be wrong in the face of either negative or very large positive effects, the Bayesian approach more strongly supports the existence of the effect, with confidence intervals that do not extend so far into the negative range. Confidence intervals are generally smaller, because the estimated effects are within our prior expectations based on study 1. Assessing the confidence of these effects against the belief that any effect size is possible would be to put ourselves back in a position of ignorance.

**Table 1:** Study 2 parameter estimates for the Bayesian regression model.

Parameter	Mean (*SD*)	95% interval
**Intercept**	-.16 (.08)	[-.32, .00]
**M. Salience**	.14 (.10)	[-.06, .34]
**Reverse cards**	.12 (.11)	[-.10, .34]
**Both manipulation**	.32 (.10)	[.12, .52]
**Sigma**	.99(.03)	[.93, 1.05]

We can reach a number of conclusions with the Bayesian approach that are more difficult from a frequentist framing. A) Our two studies have produced effect sizes within tolerance of each other. B) The effect size is likely smaller than our first study suggested; effect sizes that could produce the distributions in both studies 1 and 2 are unlikely to be larger than .3. C) despite the single threat conditions being not significant using either frequentist or Bayesian analyses, we are nonetheless more confident that an effect exists.

### Study 3

Using the same strategy in study 3, we updated our prior expectations to match a posterior distribution from study 2, blending the null and experimental models based on their evidential weight. Given that we have effect estimates for each type of meaning threat now, we estimated separate prior distributions for each condition in line with their coefficient and standard deviation. Again, the practical effect of the new priors is that effect sizes between -.05 and .35 will be interpreted as more likely.

Descriptive statistics for scores on the DV for each condition: control *N* = 47, *M* = .58, *SD* = .28; mortality salience *N* = 38, *M* = .57, *SD* = .24; cards *N* = 46, *M* = .70, *SD* = .26; both manipulations *N* = 43, *M* = .69, *SD* = .26 (8 participants were removed because the experimenter noted a problem during collection). Looking at effect sizes within the frequentist regression model, we find that mortality salience has an effect in the opposite direction as predicted *B* = -.05[-.47, .37], *p* = .83. The other two conditions are significant in the expected direction: cards *B* = .47[.07, .87], *p* = .02, both manipulations *B* = .44[.03, .84], *p* = .04. However, the cards condition is arguably an over-estimate. Given the previous studies, it is unrealistic to take the point estimate of .44 at face value as representing the true underlying effect.

**Fig 3:**
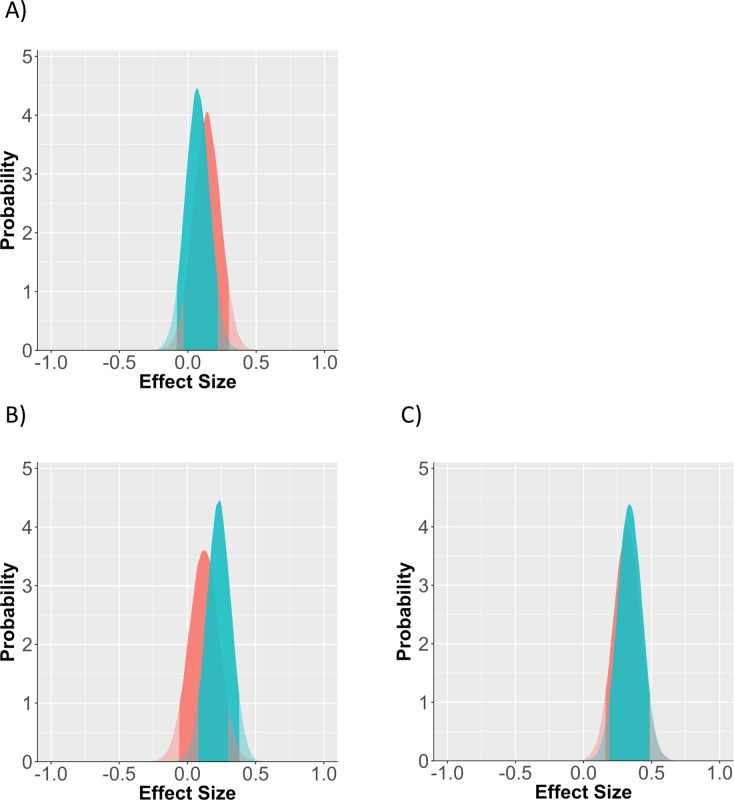
Prior vs posterior distribution for each of the 3 conditions. (A) mortality salience, (B) cards, (C) both manipulations. Red distribution is the prior probability of the effect, green is the posterior distribution which accounts for study data. Solid region represents the 95% probability window, shaded regions are outside this window.

Comparing to the Bayesian model, we find the first clear example of the two analysis strategies diverging (See Fig 3 for prior and posterior distributions, and Table 2 for parameter estimates). Despite the cards and duel threat conditions showing strong effects in the conventional analysis, the Bayesian regression estimates that a more moderately sized effect likely underlies the data, given the current data and our prior expectations. Likewise, although the mortality salience group has a lower working memory score than the control group, our estimate of the underlying effect is still positive (with a confidence tail that extends farther into the negative space). However, note also that our confidence interval of the effect has not reduced at the rate of the previous studies. Relative to the amount of data from the previous studies, the current study with its smaller sample only provided a minor contribution. In this way, it is possible to add a large number of studies with relatively small N to the analysis; smaller samples that don't match the prior distribution pose less of a direct challenge to our initial assumptions. Likewise, small samples that agree with our prior assumptions don't necessarily help us shorten our confidence intervals.

**Table 2:** Study 3 parameter estimates based on Bayesian regression model.

Parameter	Mean (*SD*)	95% interval
**Intercept**	-.16 (.08)	[-.32, .00]
**M. Salience**	.07 (.09)	[-.11, .25]
**Reverse cards**	.23 (.09)	[.05, .41]
**Both manipulations**	.34 (.09)	[.16, .52]
**Sigma**	.98 (.05)	[.89, 1.08]

### Study 4

Priors for effect sizes were updated based on the posterior distribution of study 3. Descriptive statistics on digit span scores for each condition are: Control *N* = 81, *M* = .50, *SD* = .27; mortality salience *N* = 92, *M* = .53, *SD* = .23; reversed cards *N* = 95, *M* = .59, *SD* = .19; both manipulations *N* = 80, *M* = .53, *SD* = .23 (53 participants were removed either due to technical errors that led to missing dependent variable values, for failing one of our quality checks, or because they admitted to cheating during the debriefing). The conventional analysis indicates that only the cards condition produced a significant effect: mortality salience *B* = .11[-.19, .41], *p* = .46; reverse cards *B* = .37[.08, .67], *p* = .02; both manipulations *B* = .09[-.22, .39], *p* = .58. Given what we know about past effect size estimates from these manipulations, the current confidence intervals are needlessly pessimistic when taken out of context.

**Fig 4:**
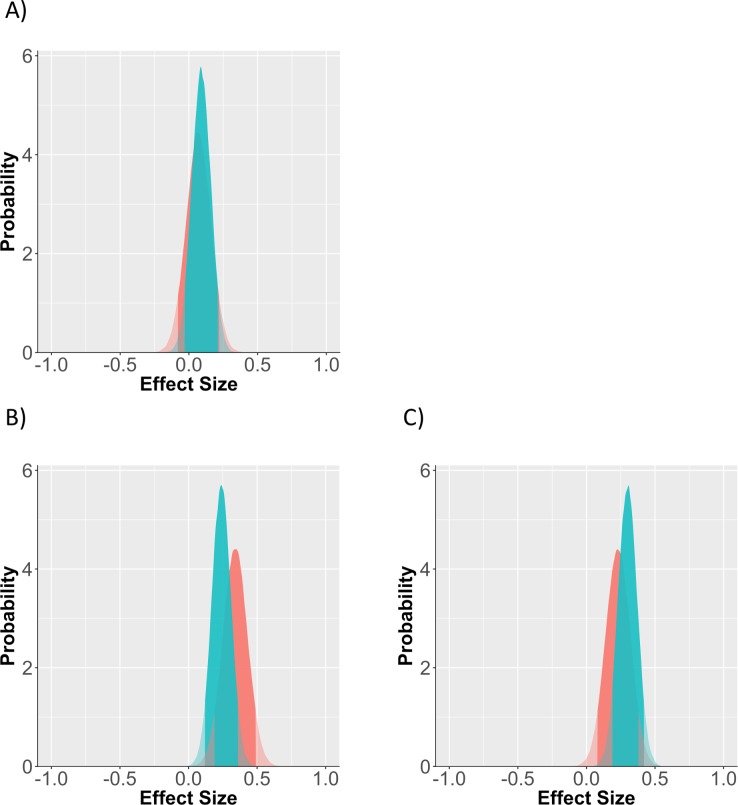
Prior vs posterior distribution for each of the 3 conditions. (A) mortality salience, (B) reversed cards, (C) both manipulations. Red distribution is the prior probability of the effect, green is the posterior distribution which accounts for study data. Solid region represents the 95% probability window, shaded regions are outside this window.

When considering the results from a Bayesian perspective, the final posterior distributions are more optimistic. Based on the current sample and evidence, in combination with our expectations for the likely window containing the effect size, both the cards condition and the dual meaning threat condition likely represent a moderate sized effect (See Fig 4 for prior and posterior distributions, and Table 3 for parameter estimates). The bulk of the probability space is also in the small and positive direction for mortality salience, though the 95% confidence interval crosses zero.

**Table 3:** Study 4 parameter estimates from Bayesian regression model.

Parameter	Mean (*SD*)	95% interval
**Intercept**	-.16 (.06)	[-.28, -.04]
**M. Salience**	.09 (.07)	[-.04, .23]
**Reverse cards**	.30 (.07)	[.16, .43]
**Both manipulation**	.24 (.07)	[.10, .38]
**Sigma**	.99 (.04)	[.92, 1.06]

### Follow-up

**Fig 5:**
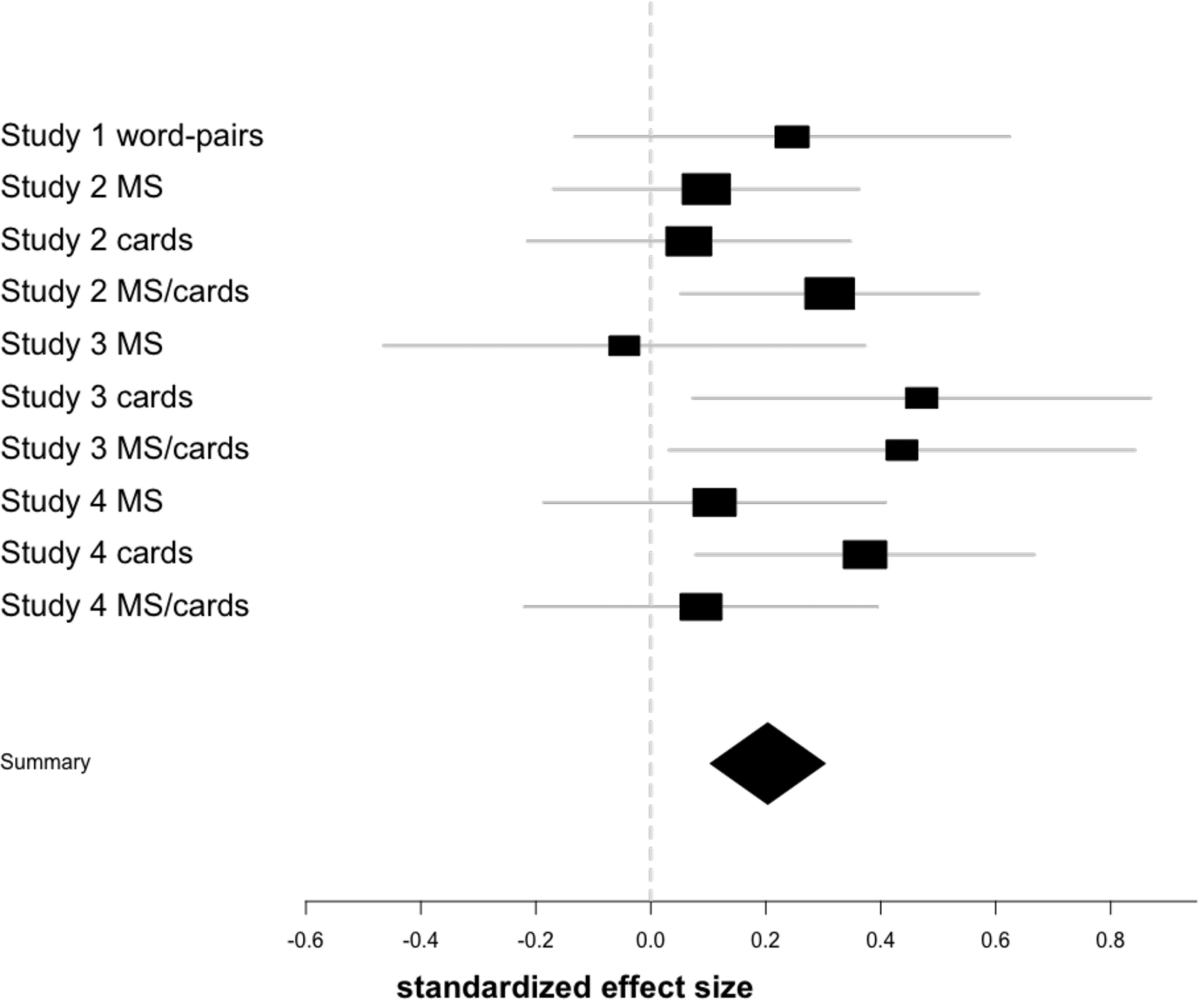
Meta-analytic forest plot of all experimental effects. Squares are positioned based on the standardized regression coefficient, size is in relation to sample size. Bars represent 95% confidence interval. The large diamond is the meta-analytic average and confidence of the true underlying effect.

The two analysis approaches lead to somewhat different conclusions in the final analysis. Although we would also conclude with frequentist statistics that a small effect likely exists based on meta-analysis (See Fig 5 for a meta-analysis), it is difficult to see that effect emerge with each study, starting with flat priors in each analysis.

Emphasizing whether our point estimate has confidence intervals that do not cross zero is also demoralizing, likely unreasonably so given the small size of the effect. For example, based on the Bayesian interpretation we are confident that the effect of the cards manipulation causes an increase somewhere between .16 and .43 standard deviations on the working memory task. However, we also know (because we defined it) that the sample these estimates were drawn from has a standard deviation of 1. It would be very easy to draw a sample that does not reveal the effect, or shows the opposite. This leads to the question of replication: What would qualify as a successful replication (or refutation of our finding) and how large a sample would one need? The answer is different for either frequentist or Bayesian thinking. From a frequentist perspective, we would like our 2-condition replication experiment to produce a significant difference. Simulating studies of *N* = 50 per condition (1 000 simulations) and increasing by 50, we can see how large a sample is needed to achieve 80% power for finding this effect (See Table 4 for parameter estimates).

**Table 4:** Power to detect the true effect of .16 - .43.

N per condition	Power	% significant but wrong	% point estimate within CI for all simulations
50	.33	.79	.50
100	.52	.36	.64
150	.75	.17	.78
200	.84	.13	.83
250	.91	.09	.85
300	.96	.05	.92

N, number of participants in each condition of 2-condition test (control vs. meaning threat); Power, percentage of simulated regressions that produce a significant effect for meaning threat; % significant but wrong, the percentage of the significant results that yielded an effect size that is outside our expected effect size range of .16 to .43; % point estimate within CI, percentage of all the simulated trials (whether significant or not) that yield a point-estimate of the effect within our posterior expectations of .16 - .43. Each sample size was simulated 1000 times.

First thinking about conventional replication. With a sample of 50 participants per condition (what used to be the gold standard) we would have 33% power to detect the effect. However, nearly 80% our significant effect size estimates would be outside the range of the real effect, mostly over-estimating the effect due to chance sampling fluctuations. To achieve 80% power, we would need just under *N* = 200 per condition (400 participants for a 2-condition study), though even then more than 10% of our significant results will have over- or under-estimated the effect. But then again, do we need to replicate in a single study that the effect is "not zero"? This is an uninteresting and actually far more vague prediction than "the true effect is within .16 and .43". The latter prediction is more precise, and theoretically more meaningful (i.e. we are claiming the effect exists, and that we are quite confident that it is fairly small to moderate in size). A better bar for replicating would be a study that produces a point-estimate of the effect size within our confidence interval. While in the case of our results, both approaches would require just under *N* = 200 per condition, focusing on the effect size will keep the required sample at roughly this size even for smaller effects, while the sample needed for significance can increase dramatically. Additionally, it lets us shift the conversation away from not-zero towards "how sure are we of the effect size"? At that sample size, estimates close to zero give us pause that perhaps the effect is not real, and effects larger than .39 suggest that perhaps population or methodological factors may moderate the effect. In either case, the new data can be used to update our priors, helping us to shift and adjust our confidence appropriately.

## Discussion

Four studies investigated the relationship between uncertainty and working memory capacity. In the first study, we measured performance on a word span (working memory) task after participants were exposed to either senseless or sensible word pairs. The results of this study suggested either a small effect, or no effect, of uncertainty on working memory capacity. In Study 2, we detected a similarly small effect using an Mturk sample. We employed different manipulations including a blackjack game with reversed-cards, a mortality salience prime, and a condition that combined both uncertainty primes (dual meaning threat). Study 3 employed the same manipulations as Study 2, but introduced a new DV; a digit span task in which participants recalled long strings of numbers. The mortality salience condition had an effect in the opposite direction, and the other two conditions were significant in the expected direction. Altogether, the findings from the third study were consistent with a small positive effect. Study 4 was a direct replication of Study 3 using an Mturk sample, in which a moderate effect of the reversed cards condition and the dual meaning threat on working memory capacity. Taken together, we are reasonably confident that the true effect size for the reverse-cards manipulation, and the two uncertainty manipulations together, are small to moderate. We are less confident about the mortality salience condition, and are not confident that presenting the two uncertainty manipulations together (cards and mortality salience) makes the effect stronger. Ultimately, we were able to conclude that we are dealing with an effect that is non-zero but discouragingly difficult to detect. We advise that future studies use a much larger sample size of N = 200 per group to overcome this difficulty.

Our interpretation is that the importance of these studies lies in their ability to provide theoretical context for a phenomenon observed in a diverse set of literatures; namely, that people experience an increase in their ability to learn and process information when they encounter an uncertain event (see [17,36,37], see also [38,39]). Specifically, we are able to conclude that working memory capacity is one executive function that may contribute to this increase. Therefore, we posit that the findings from the present set of studies represent an important new direction in uncovering the cognitive mechanisms that allow people to learn more about their environment when confronted with uncertainty.

Our findings also shed light on some ambiguities in the threat compensation literature. Because we used a diverse set of uncertainty manipulations (mortality salience, reverse-colored playing cards, and senseless word pairs) we may conclude that counter to other theories in the threat compensation literature (see [17,37]) this pattern-seeking behavior is not specific to solving the source of uncertainty; rather, it is a nonspecific attempt to re-establish order in the environment. While there is still some doubt about the strength of the mortality salience manipulation, our other manipulations—which are in fact harder to explain with alternative theories because they operate implicitly—show convergent results.

It is important to note that our findings do not suggest that uncertainty always leads to increased working memory capacity. Indeed, there is reason to believe that people resolve threats to certainty in many different ways. Greater attentional control is a feature of abstraction, which is only one of the proposed mechanisms by which people reduce the negative arousal associated with uncertainty. We speculate that the size of the effect may reflect a general preference for other anxiety-reducing strategies; for example, people have been known to affirm existing schemas in order to compensate for perceived meaninglessness in another domain (e.g., [7,9]). A future study may involve multiple uncertainty-reducing tasks, and a comparison of the effects obtained for each. Future studies should also determine if anxiety is indeed the source of all of these behaviours. More narrowly, future research should determine if anxiety mediates the relationship between uncertainty and working memory, using indicators of autonomic arousal such as skin conductance.

There are a number of limitations to the studies presented here. The small effect size suggests that the exact mechanism by which all of these changes in attention occur is still unknown. Indeed, there is no firm evidence that the many cognitive and attitudinal changes in processing that follow threats to meaning can be attributed to working memory capacity and not a related mechanism. For example, though we find evidence for changes in working memory in the present research, the working memory tasks we employ may be somewhat idiosyncratic, measuring constructs that are related to, but distinct from, working memory. That is, both the digit span task (used in studies 3 and 4) and the operation span task (used in studies 1 and 2) require that participants retrieve information from memory rather than engage in simple attentional control. On the other hand, the most common definition of working memory is a construct that involves multiple mechanisms for organizing and manipulating information [88] as well as retrieving information from secondary memory [89,90], these task-related idiosyncrasies become less of a concern (indeed, they may provide the best test of our hypothesis that discrepancies affect working memory, rather than smaller dissociable mechanisms that underlie working memory). Furthermore, both the digit span task and the operation span task represent the most commonly-used and straightforward measures of working memory capacity [91,92,93] indicating that at the very least, these tasks reflect the underlying construct reasonably well. Therefore, we have some reason to suspect that working memory, as opposed to related constructs, is the mechanism at play in the current research, although we acknowledge that it remains to be seen whether the same pattern of results would be found for all measures of working memory.

Another concern with the present research is that our small effects may indicate that there are untested moderators dampening this effect. To address the latter possibility, we suggest that future studies determine if individual differences moderate this relationship; for example, differences in approach and avoidance motivation, which have been found to predict the strength of responses to threat (e.g., [94,95]).

We also acknowledge that the present studies do not provide imaging or psychophysiological data to speak to our proposed mechanism: activation in the ACC caused by threat, leading to increased working memory capacity. Future research employing fMRI or EEG could determine if ACC activation is indeed implicated in the relationship between threat and working memory capacity.

It is also unclear how well our findings would generalize to other samples. However, we managed to find similar effects among Canadian undergraduates and an American sample over Mturk. We therefore speculate that the results generalize to diverse populations, although we suggest that future studies use non-Western samples as well. It is also difficult to determine if working memory capacity is increased consciously or unconsciously. An unconscious account fits better with past results of meaning threats enhancing implicit pattern learning [38,39]; however, it remains possible that some people may have explicit awareness of their greater attentional focus. Future studies can include measures of attentional control that have been known to be processed explicitly rather than implicitly, or vice versa.

Despite these limitations, our findings serve as evidence that uncertainty leads people to pay more attention to information in the environment. In uncovering one of the mechanisms governing this effect; attentional control improving working memory; we provide some direction for the study of meaning-making and how people navigate an increasingly confounding world.
